# Guiana Dolphin Unusual Mortality Event and Link to Cetacean Morbillivirus, Brazil

**DOI:** 10.3201/eid2407.180139

**Published:** 2018-07

**Authors:** Kátia R. Groch, Elitieri B. Santos-Neto, Josué Díaz-Delgado, Joana M.P. Ikeda, Rafael R. Carvalho, Raissa B. Oliveira, Emi B. Guari, Tatiana L. Bisi, Alexandre F. Azevedo, José Lailson-Brito, José L. Catão-Dias

**Affiliations:** Universidade de São Paulo, São Paulo, Brazil (K.R. Groch, J. Díaz-Delgado, J.L. Catão-Dias);; Universidade do Estado do Rio de Janeiro, Rio de Janeiro, Brazil (E.B. Santos-Neto, J.M.P. Ikeda, R.R. Carvalho, R.B. Oliveira, E.B. Guari, T.L. Bisi, A.F. Azevedo, J. Lailson-Brito)

**Keywords:** *Sotalia guianensis*, Paramyxoviridae, morbillivirus outbreak, die-off, dolphin, immunosuppression, pathology, marine mammals, viruses

## Abstract

During November–December 2017, a mass die-off of Guiana dolphins (*Sotalia guianensis*) began in Rio de Janeiro, Brazil. Molecular and pathologic investigations on 20 animals indicated that cetacean morbillivirus played a major role. Our findings increase the knowledge on health and disease aspects of this endangered species.

Cetacean morbillivirus (CeMV; family Paramyxoviridae) is a highly infectious pathogen responsible for numerous cetacean mass die-offs worldwide. Currently, there are 3 well-characterized strains (*1*), the porpoise morbillivirus, the dolphin morbillivirus, and the pilot whale morbillivirus, and 3 less-known strains, including the novel Guiana dolphin strain (GD)–CeMV, recently identified in a single specimen from Brazil (*2*). CeMV was detected in Ireland, England, and the Netherlands in 1988–1990 (*3**,**4*), when the porpoise morbillivirus strain was identified in a small number of stranded harbor porpoises (*Phocoena phocoena*). Since then, CeMV has been implicated as the causal agent of numerous outbreaks and also endemic, sporadic deaths involving multiple cetacean species throughout the North Sea, north Atlantic Ocean, Mediterranean Sea, Black Sea, Indian Ocean (Western Australia), and Pacific Ocean (Hawaii, Japan, and Australia) (*1*).

To date, no epizootics linked to CeMV causing the death of large numbers of marine mammals has been detected in the South Atlantic. A Guiana dolphin (*Sotalia guianensis*) stranded in Espírito Santo, Brazil, which tested positive for CeMV by reverse transcription PCR (RT-PCR) and immunohistochemistry, has been the only confirmed fatal case in South Atlantic cetaceans (*2*). We describe the results of pathologic and molecular investigations on 20 deceased Guiana dolphins in the onset of the ongoing unusual mortality event in Rio de Janeiro, Brazil.

## The Study

During November–December 2017, a unusual mortality event involving 56 Guiana dolphins began in Ilha Grande Bay, Rio de Janeiro (Brazil; 23°4′45′′–23°13′38′′S, 44°5′30′′–44°22′28′′W). This area is a relatively well-preserved ecosystem, and Guiana dolphin population census size in this area was estimated at ≈900 animals (*5*). Stranding occurrence for the same period in previous years ranged from 0 to 3 specimens. During this event, carcasses were recovered adrift or washed ashore. We performed necropsies on 20/56 (37.7%) Guiana dolphins and recorded epidemiologic and biologic data (Table 1).

**Table 1:** Individual epidemiologic stranding data and biologic data of 20 Guiana dolphins stranded or retrieved from Ilha Grande Bay, Rio de Janeiro, Brazil, November 2017*

No.	Date found	Location coordinates	Sex	Body length, cm	Age class	Body condition	Decomp status	Main gross findings	CeMV RT-PCR, tissue/result
1	9	−23.16738, −44.13948	F	177	Adult	Poor	Fr	Lactation; verminous pneumonia; pulmonary edema; mediastinal empyema; gastrointestinal petechiae, gastrointestinal parasitosis; absence of ingesta	Lung/pos,† brain/pos, spleen/pos
2	14	−23.01327, −44.44241	M	94	Calf	Moderate	Fr	Cyanotic mucous membranes; pulmonary edema; hepatic lipidosis; gastrointestinal petechiae	Lung/pos,† liver/pos
3	14	−23.15123, −44.32286	F	124	Juvenile	Poor	MA	Pulmonary edema; gastric ulcers; absence of ingesta	Lung/pos,† liver/pos
4	23	−23.0319, −44.54259	ND	71.5	Calf	ND	AA	ND (AA)	Lung/pos†
5	23	−23.03725, −44.55784	ND	189	Adult	ND	AA	Verminous pneumonia	Lung/pos,† liver/pos
6	23	−23.08996, −44.35695	M	160	Juvenile	Poor	MA	Verminous pneumonia; pulmonary edema; absence of ingesta	Lung/pos,† brain/pos, spleen/pos
7	24	−23.00963, −44.35695	ND	170	ND	ND	AA	ND (AA)	Kidney/pos†
8	24	−22.07896, −44.23156	ND	132	Juvenile	ND	AA	Black stained ingesta (plastic)	Liver/neg, spleen/neg
9	26	−23.03688, −44.55140	M	167	Juvenile	ND	AA	ND (AA)	Kidney/neg
10	25	−23.04786, −44.57191	M	123	Juvenile	Good	MA	Proliferative pleuritis and peritonitis; gastrointestinal parasitosis; absence of ingesta	Lung/pos,† brain/pos, spleen/pos
11	25	−23.03637, −44.55041	F	123	Juvenile	ND	AA	ND (AA)	Lung/pos,† brain/pos, spleen/pos
12	27	−23.01980, −44.44088	F	124	Juvenile	ND	MA	ND (AA); absence of ingesta	Lung/pos,† brain/pos, spleen/pos
13	27	−23.04542, −44.59536	F	142	Juvenile	ND	AA	Verminous pneumonia; gastrointestinal parasitosis; absence of ingesta	Lung/pos, brain/pos†
14	28	−23.16538, −44.63874	M	118	Juvenile	ND	AA	ND (AA); absence of ingesta	Lung/pos, brain/pos, spleen/pos†
15	28	−23.1325, −44.62048	F	182	Adult	ND	AA	Hydrothorax and ascites; verminous pneumonia; absence of ingesta	Lung/pos,† brain/pos, spleen/pos
16	28	−23.12665, −44.622	M	89	Calf	ND	AA	Autolysis; absence of ingesta	Lung/neg, spleen/ neg
17	29	−23.11585, −44.66409	F	170	Adult	ND	MA	Pulmonary edema; absence of ingesta	Lung/pos,† spleen/pos
18	29	−23.12927, −44.66989	M	156	Juvenile	Moderate	Fr	Fishing gear lesions; hydrothorax and ascites; verminous pneumonia; pulmonary edema; hemopericardium; gastroenteritis; gastrointestinal parasitosis; urinary bladder petechiae; pterygoid and tympanic bullae trematodiasis	Lung/neg, spleen/neg
19	29	−23.12726, −44.67302	F	144	Juvenile	Good	Fr	Fishing gear lesions; hydrothorax and ascites; verminous pneumonia; pulmonary edema; gastric ulcer; gastrointestinal petechiae	Lung/neg, spleen/neg
20	30	−23.07919, −44.55559	M	125	Juvenile	ND	AA	ND (AA); absence of ingesta	Lung/pos,† brain/pos

*Collection period was November 9–December 29, 2017. AA, advanced autolysis; decomp, decomposition; Fr, fresh; MA, moderate autolysis; ND, not determined; neg, negative; no., animal no.; pos, positive; RT-PCR, reverse transcription PCR. †Amplified fragment sequenced.

We collected representative tissue samples of major organs and fixed them in 10% neutral buffered formalin or froze them at −80°C. For PCR analysis, we extracted viral RNA from frozen lung, brain, spleen, liver, and kidney (Table 1) using Brazol Reagent (LGC Biotecnologia Ltda, São Paulo, Brazil), according to the manufacturer’s instructions. We used random primers and M-MLV Reverse Transcription Kit (Invitrogen, Life Technologies, Carlsbad, CA, USA) to synthesize cDNA. We performed amplification using primers targeting highly conserved fragments of the phosphoprotein (P) gene (*6*) and RNA-dependent RNA polymerase protein coded by the L gene (primers RES-MOR-HEN) as previously described (*2**,**7*). 

**Figure 1:**
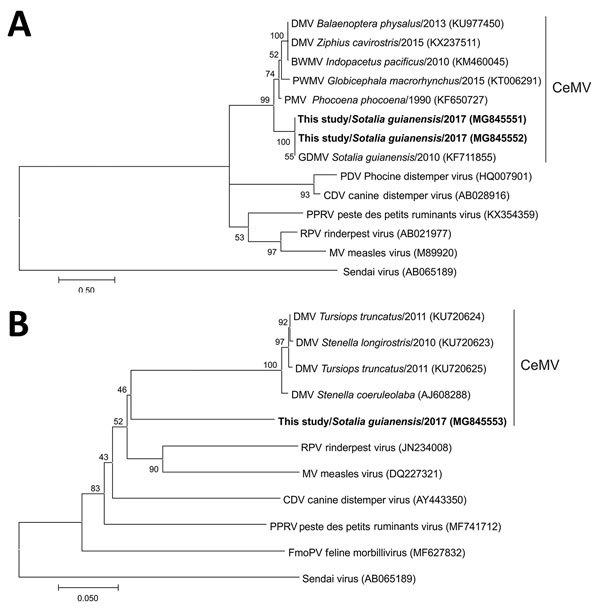
Phylogenetic tree showing partial sequence of A) morbillivirus phosphoprotein and B) large protein genes of cetacean morbillivirus (CeMV) isolates found in stranded Guiana dolphins (*Sotalia guianensis*) from Rio de Janeiro, Brazil (bold), 2017, and those of other previously described morbilliviruses. Sendai virus was added as an outgroup. Trees were generated by the maximum-likelihood method (A) and neighbor-joining method (B); bootstrap values (1,000 replicates) are indicated at the internal nodes. For comparison, recognized CeMV strains were included when available. Sequence names are followed by species, year of stranding (when available), and GenBank accession number. Scale bars indicate nucleotide substitutions per site. PMV, porpoise morbillivirus; DMV, dolphin morbillivirus; BWMV, beaked whale morbillivirus; PWMV, pilot whale morbillivirus.

We detected CeMV genome in 15/20 (75%) animals for the P gene and 6/6 (100%) animals for the L gene. We sequenced amplified products and compared them with sequences of CeMV using blastn (http://blast.ncbi.nlm.nih.gov/Blast.cgi). We obtained identical sequences for the L gene, and 2 sequences with variation in 1 nucleotide position for the P gene. Sequencing of 405-bp amplified fragments of the CeMV P gene revealed 99%–100% identity to GD-CeMV (*2*) and 78%–82% identity with other CeMV strains. A 443-bp amplified fragment of the CeMV L gene revealed 74%–75% identity to CeMV and other morbillivirus species. Partial P and L gene sequencing and analysis using MEGA7 (http://megasoftware.net/) corroborate that the GD-CeMV strain differed from other morbilliviruses and represented a distinct lineage (Figure 1).

**Figure 2:**
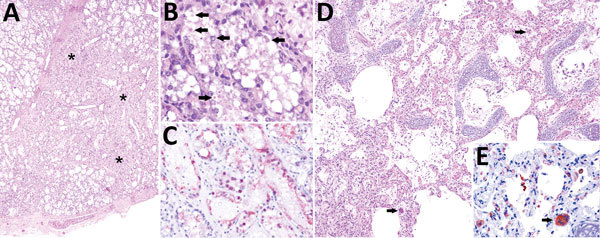
Cetacean morbillivirus–associated histopathologic findings in 2 Guiana dolphins (*Sotalia guianensis*), a female adult (case 1, panels A–C) and a male calf (case 2, panels D–E). A) The mammary gland parenchyma is focally disrupted by lymphohistiocytic inflammatory cells (not visible at this magnification) associated with collapsed and lost acini, and mild fibrosis (asterisks). Original magnification ×40; hematoxylin and eosin (H&E) stain. B) Swollen and degenerating mammary acinar epithelial cells have numerous intracytoplasmic and intranuclear inclusion bodies (arrows). Original magnification ×200; H&E stain. C) Degenerating and sloughed mammary acinar epithelial cells have intense granular cytoplasmic and intranuclear immunolabeling, identified by immunohistochemistry (IHC) for canine distemper virus (CDV), known to cross react with cetacean morbilliviruses. D) Pulmonary area displaying interstitial pneumonia with mildly thickened alveolar septa and alveoli containing proteinaceous edema, scattered fibrin strands, and small numbers of pleocellular inflammatory cells including occasional syncytia (arrows). Original magnification ×100; H&E stain. E) Degenerating and necrotic type I pneumocytes, sloughed and adhered type II pneumocytes, alveolar and septal macrophages, syncytia (arrow) and circulating (intravascular) mononuclear cells display intense immunolabeling. Original magnification ×400; IHC for CDV.

For histologic examination, we embedded formalin-fixed tissues in paraffin wax, processed them as routine, and stained them with hematoxylin and eosin. We recorded detailed histopathologic findings of 6 animals positive for CeMV by RT-PCR (Table 2). One specimen had lesions consistent with CeMV infection, including marked multifocal, subacute bronchointerstitial pneumonia with type II pneumocyte hyperplasia, syncytia, and scattered intraepithelial, intranuclear, and intracytoplasmic inclusion bodies (INCIBs); mild to moderate multifocal histiocytic and lymphoplasmacytic mastitis with necrosis and epithelial INCIBs (Figure 2, panels A–C); and multicentric lymphoid depletion. In addition, most animals had moderate to severe verminous bronchopneumonia and pleuritis with morphologic evidence of pulmonary arterial hypertension, multicentric eosinophilic and necrotizing lymphadenitis, and chronic aortic endarteritis by adult nematodes and pulmonary endarteritis by migrating larval nematodes histomorphologically compatible with *Halocercus brasiliensis* (*8*). Other common findings included moderate to poor body condition and lack of ingesta with small amounts of feces. Two (10%) of the 20 animals (which were negative for CeMV by RT-PCR) showed typical external net markings and multiorgan acute hemodynamic alterations (congestion, edema, and hemorrhage) supporting asphyxia due to bycatch as the cause of death.

**Table 2:** Summary of histopathologic findings for 6 Guiana dolphins recovered from Ilha Grande Bay, Rio de Janeiro, Brazil, 2017*

No.	Tissue	Histopathologic findings
1†	Lung	Marked, multifocal chronic bronchointerstitial pneumonia and proliferative pleuritis with sclerosis, type II pneumocyte hyperplasia, syncytia/multinucleated cells, rare INCIBs, calcified nematode debris and edema; multifocal tunica media hypertrophy/hyperplasia
	Mammary gland	Mild to moderate, multifocal, chronic lymphoplasmacytic and histiocytic mastitis with acinar ectasia, inspissated secretion, scattered necrosis, ceroid pigment and moderate INCIBs in epithelium
	Heart	Mild, focal, subacute fibrinous pericarditis; mild, multifocal myocardial fibrosis
	Kidney	Mild, multifocal, chronic membranous glomerulonephritis with glomerulocysts, tubular proteinosis, protein casts, and scattered tubuloepithelial necrosis
	Pulmonary lymph node	Mild, multifocal, chronic nodular eosinophilic and necrotizing lymphadenitis with fibrosis and hemosiderosis; diffuse lymphoid depletion
	Mediastinal lymph node	Mild, multifocal, chronic eosinophilic lymphadenitis; diffuse congestion
	Spleen	Diffuse congestion and multifocal, acute capsular hemorrhage; extramedullary hematopoiesis
	Adrenal	Mild, multifocal, acute corticomedullary hemorrhage
	Aorta	Mild, segmental, chronic proliferative endarteritis
	Liver	Moderate, multifocal, chronic bile duct adenomatous hyperplasia
	Uterus	Moderate, multifocal, chronic arteriosclerosis and arterial elastosis
	Glandular stomach	Mild, diffuse mucosal hyperplasia; multifocal arterial tunica media hypertrophy/hyperplasia
2‡	Lung	Mild, multifocal, acute interstitial pneumonia associated with marked alveolar edema, hemorrhage and alveolar histiocytosis, syncytia/multinucleated cells and keratin spicules
	Kidney	Mild, multifocal, acute tubular degeneration and necrosis; mild, multifocal, acute tubular proteinosis and protein casts; marked, focal, acute perirenal hemorrhage
	Pulmonary lymph node	Moderate, diffuse cortical and paracortical lymphoid depletion with scattered lymphocytolysis
	Prescapular lymph node	Diffuse congestion with focal acute hemorrhage and erythrophagocytosis; sinus vascularization
	Spleen	Moderate, diffuse, lymphoid depletion with sinus histiocytosis; extramedullary hematopoiesis
	Heart	Moderate, multifocal, acute subendocardial and epicardial hemorrhage
	Adrenal	Marked, multifocal, acute cortico-medullary hemorrhagic necrosis
	Esophagus	Focal acute hemorrhage in serosa
	Urinary bladder	Edema and focal acute hemorrhage in serosa
	Penis	Urethral luminal hemorrhage with single cell epithelial necrosis/apoptosis
	Cerebrum, cerebellum	Diffuse leptomeningeal congestion and perivascular edema in neuroparenchyma
10§	Lung	Mild to moderate, multifocal, chronic suppurative bronchopneumonia with adult nematodes (*Halocercus* sp.); multifocal alveolar, bronchiolar and bronchial mineralization
	Keratinized stomach	Mild, focal, chronic proliferative gastritis
	Skeletal muscle	Scattered acute hyaline myocyte degeneration and necrosis
11	Ascending aorta	Marked, segmental, chronic fibrosing and proliferative endarteritis with chondroid metaplasia and calcification; moderate, focal, chronic granulomatous periarteritis; mild, multifocal intimal fibroelastosis
	Aortic (semilunar) valve	Mild, multifocal, chronic intimal/endocardial fibroelastosis
12¶	Keratinized stomach	Mild, focal, chronic mononuclear gastritis
13#	Lung	Marked, multifocal, chronic suppurative to pyogranulomatous bronchopneumonia with bronchial/olar sclerosis, adult and larval nematodes (*Halocercus* sp.) and moderate, multifocal, chronic proliferative and fibrosing (villous) pleuritis; marked, multifocal, chronic tunica media arterial hypertrophy/hyperplasia with stenosis
	Skin	Mild, multifocal, chronic irregular epidermal hyperplasia
	Pyloric stomach	Moderate, focal, chronic granulomatous gastritis with numerous trematode ova (compatible with *Pholeter gastrophilus*)

*INCIBs, intranuclear and intracytoplasmic inclusion bodies; no., animal no. †No significant lesions were observed for large intestine, thyroid, skin, trachea, cerebrum, cerebellum, spinal cord, or skeletal muscle. ‡No significant lesions were observed for keratinized and pyloric stomach, tongue, aorta, small intestine, pancreas, or trachea. §Unable to observe lesions in liver, spleen, kidney, testicle, trachea, small and large intestine, skin, periaortic lymph node, cerebrum, or cerebellum because of advanced autolysis of animal. ¶Unable to observe lesions in ovary, skin, liver, skeletal muscle, heart, kidney, spleen, large intestine, bladder, lymph node, or adrenal gland because of advanced autolysis of animal. #Unable to observe lesions in small intestine, liver, skeletal muscle, adrenal gland, bladder, kidney, or heart because of advanced autolysis of animal.

We performed immunohistochemistry studies using a monoclonal antibody against the nucleoprotein antigen of canine distemper virus (CDV-NP mAb; VMRD Inc. Pullman, WA, USA), as described (*2*). In lung tissue sections (cases 1, 2, 11, and 13), we evaluated number and distribution of immunopositive cells and immunolabeling intensity. Lung samples from all animals tested showed widespread and intense immunolabeling in bronchial, bronchiolar, and alveolar epithelium, alveolar macrophages, and syncytia (Figure 2, panels D,E).

In this investigation, typical histopathologic findings consistent with CeMV were evident in 1 animal, indicating a systemic infection. Although chronic bronchointerstitial pneumonia and multicentric lymphoid depletion observed in most animals are common findings in CeMV-infected cetaceans, these lesions were considerably overlapped by *H. brasiliensis* endoparasitosis. The pathologic signatures of GD-CeMV remain unknown. No other CeMV strain has been described in the South Atlantic Ocean. In subacute and chronic CeMV presentations, fatalities are often ascribed to secondary infections (e.g., toxoplasmosis, aspergillosis) (*9**,**10*). In our cohort, autolysis precluded microscopic examinations in some animals, so we could not draw further pathologic conclusions. Nonetheless, moderate to severe parasitosis by *H. brasiliensis* likely accounted for severe illness in most cases. Intense viral replication in the mammary acinar epithelium in a lactating female may imply a vertical transmission route, in addition to the horizontal aerogenous and direct contact routes (*10*). Therefore, future pathologic and epidemiologic studies in the South Atlantic should consider vertical transmission. Two cases from this cohort were bycaught, further supporting the multifactorial nature of the ongoing unusual mortality event.

The Guiana dolphin is a coastal and estuarine delphinid endemic from southern Brazil to Central America and one of the most threatened South Atlantic cetaceans, for which recent studies demonstrate severe population decline (*11*). Because of its near-shore distribution and site fidelity (*12*), the Guiana dolphin is susceptible to the effects of human activities (e.g., habitat degradation, chemical pollution, noise, and bycatch) (*13*). Many intricate and complex anthropic and natural factors interplay and modulate the decline of species. Human activities are by far the major threat and cause for decimation of cetacean populations (*14*); however, natural factors such as highly infectious pathogens, e.g., CeMV, may drive decimating events in susceptible hosts (*15*).

## Conclusions

We provide compelling molecular and pathologic evidence associating GD-CeMV infection with the ongoing Guiana dolphin mass die-off near Rio de Janeiro, Brazil. As of January 2018, this event had resulted in the deaths of >200 Guiana dolphins in southern Rio de Janeiro state, and the deaths appeared to be extending southward. The environmental consequences and conservation effects, coupled with the anthropogenic threats, are expected to be dramatic. The factors underlying the die-off are being investigated, but our results indicate that GD-CeMV plays a major contributory role. Our findings increase the body of knowledge on health and disease aspects of this endangered species. 
